# Sexuality in Nigerian older adults

**DOI:** 10.11604/pamj.2015.22.315.7617

**Published:** 2015-12-02

**Authors:** Adeoti Adekunle Olatayo, Ojo Osaze Kubwa, Ajayi Ebenezer Adekunle

**Affiliations:** 1 Ekiti State University Teaching Hospital, Ado-Ekiti, Ekiti State, Nigeria; 2 General Hospital, Abuja, Nigeria

**Keywords:** Ageing, sexuality, Nigeria, sub-Saharan Africa

## Abstract

**Introduction:**

Oftentimes the older adults are assumed to be asexual as few studies explore into the sexuality of this age group worldwide and even in Nigeria. It is an important aspect of quality of life which is often neglected by people in this age group, attending physicians and the society as a whole. The study was aimed at determining the perception of older adults about sexuality, identify the factors that could militate against sexuality and fill any void in information in this regard.

**Methods:**

Descriptive study conducted in one hundred older adults. A semi-structured questionnaire was administered to consenting participants between 1^st^ of September 2013 and 31^st^ of March 2014.

**Results:**

Mean age of respondents was 66.42± 5.77 years. Seventy-eight percent of the male respondents considered engaging in sexual activity as safe compared to 45.8% of the female respondents. More of the women (33.3%) regarded sexuality in the older adults as a taboo when compared to the men (5.4%). However, the men were more favourably disposed to discussing sexual problems than the women with their spouses (42% vs 20%) and Physicians (23.2% vs 0.0%). Major factors responsible for sexual inactivity were participants’ medical ailments (65%), partners’ failing health (15%) as well as anxiety about sexual performance (25%) in the men and dyspareunia (25%) in women.

**Conclusion:**

There is an urgent need to correct the misconception about sexuality in this age group especially among the women and for the physicians to explore the sexual history of every patient.

## Introduction

Populations around the world are aging and Nigeria is no exception [1, 2]. The projected population of Nigerians aged sixty years and above by the year 2025 is six percent which is rather substantial for the most populous black nation. This rapid demographic transition towards aging population is due to government's increasing interest in the populace vital statistics as a measure of the quality of healthcare however with less emphasis on their sexuality [2, 3]. Sexuality as a topic for discussion is often circumvented, even among couples, and many times, patients find it difficult to raise the topic before their doctors. Some have suffered in silence while others believed that it should be kept sacred and secret, an issue not to be discussed outside the confines of the home. With an increasingly aging population, there is an amplified need to address the sexuality of older adults, which has the likelihood of being ignored by the patients themselves, given that they could be overwhelmed by their health status and also their socio-economic burdens. The sexual function of the older adults is an important aspect of quality of life which cannot be overlooked [4]. Previously, sexual activity was said to be restricted to procreation and it was presumed to cease once reproduction was over [5]. Several reasons have been adduced to be responsible for the declining sexuality in the older age group, some of which are the lack of interest, loss of a partner, medical and mental disabilities as well as financial constraints [6, 7]. Furthermore, the society seems not to encourage sexual relationship in the older adults as very few people could imagine their parents or grandparents still engaging in sexual activity. Research into this topic among Nigerians has limited models, with the few publications in this regard emphasizing mostly the sociological import [8, 9]. It is therefore imperative to examine the perception of this group about sexuality and identify reasons that could militate against sexual activity in the older adults.

## Methods

The study was conducted in Wuse General Hospital, Abuja in Nigeria among patients who were 60 years and above [10]. It is a descriptive study conducted between 1^st^ of September 2013 and 31^st^ of March 2014. Consenting participants were recruited from the medical outpatients and inpatients units of the hospital. A convenience sample of one hundred consenting older adults were recruited consecutively during the study period. A semi-structured questionnaire was designed to address socio-demographic characteristics, co-morbid health conditions, perception about sexual activity, frequency of sexual activity and factors responsible for the sexual dysfunction. This questionnaire was filled by the participants or a trained research assistant for those who could neither read nor write. Consenting individuals above this age who have medical, surgical or psychological disorders were included in the study. Individuals with severe dementia and previous stroke as well as non-consenting and those less than 60 years of age were excluded. Confidentiality was ensured as a written informed consent was obtained from the respondents. Ethical approval was also obtained from the institution's ethical committee with protocol approval number FHREC/2013/01/23/05-07-13. The obtained data was analyzed using statistical software SPSS version 20. Categorical variables were presented in frequencies and percentages whereas continuous variables were expressed as means± standard deviation. Comparisons of quantitative variables were performed using Pearson's chi-square test and the P-value of less than 0.05 was considered statistically significant.

## Results

**Figure 1:**
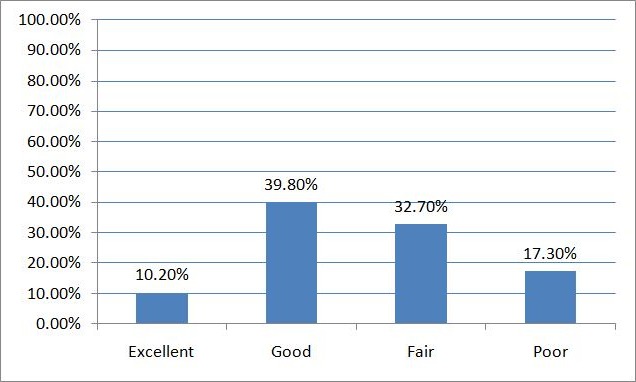
Respondents’ self-assessment of health status

**Figure 2:**
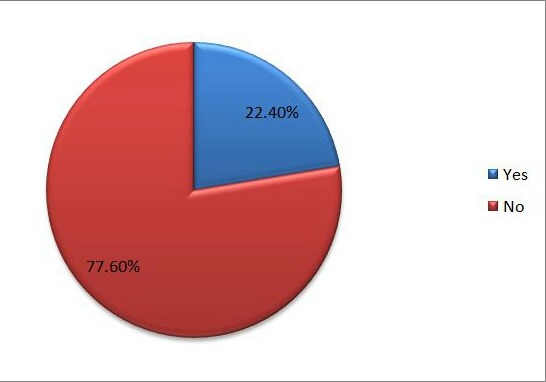
Partners’ complaints about respondents’ sexual inadequacies

One hundred subjects were recruited for the study within the age range of 60 to 83 years (mean age of 66.42± 5.77 years). Males accounted for 76% of the respondents. The majority of the respondents were retired but still actively engaged (Table 1). Common medical conditions in the older adults were systemic hypertension (61%), diabetes mellitus (30%) and arthritis (21%). The self-assessment of their current health status by respondents was excellent (10.2%), good (39.8%), fair (32.7%) and poor (17.3%) respectively as shown in Figure 1. A higher proportion of the men in this study still engaged in sexual activity which they also found pleasurable when compared to the females. The majority of the men (78.9%) considered sexual activity in this age group to be safe compared to a lower percentage in the women (45.8%). On the contrary, more women regarded sex in that age group as a taboo (33.3% vs 5.4%) and less likely to consider themselves as having sexual dysfunction (4.2% vs 28%) as shown in Table 2. Overall, a greater proportion of respondents had no complaints about their sexual performance by their partners as shown in Figure 2. Men are more favourably disposed to discussing sexual problems than the women with their spouses (42% vs 20%) and doctors (23.2% vs 0.0%). Although, more of the respondents (both male and female) still have the desire for sexual activity (74.6% vs 46.7%, chi-square= 7.891, p-value = 0.021) there is a reduced likelihood of achieving orgasm (57.7% vs 13.3%, chi-square = 3.885, p-value = 0.084). The majority of the male respondents were still able to achieve an erection (70.4%) while ejaculation was only reported in 57.7%. The major factors responsible for sexual dysfunction among the respondents were participants’ medical ailments (65%), partners’ failing health (15%) and anxiety about their sexual performance (25%). Anxiety about sexual performance was mainly identified in the men while dyspareunia was reported in women (25%).

**Table 1:** Socio-demographic characteristics of respondents

AGE	Male N = 76	Female N = 24	P value
Respondents’ age	66.50±5.45 years	66.17±5.66 years	0.80
Spouses’ age	53.51±7.80 years	72.02±12.08 years	<0.001
EDUCATION	Male N = 73 (%)	Female N = 24 (%)	P value
Primary	22 (30.1)	12 (50.0)	<0.0001
Secondary	26 (35.6)	1 (4.2)	
Tertiary	14 (19.2)	4 (16.7)	
Postgraduate	11 (15.1)	1 (4.2)	
None	0 (0.0)	6 (25.0)	
TRIBE	Male N = 74 (%)	Female N = 24 (%)	P value
Yoruba	7 (9.4)	2 (8.3)	0.623
Ibo	60 (81.2)	18 (75.0)	
Hausa	7 (9.4)	4 (16.7)	
OCCUPATION	Male N = 74 (%)	Female N = 20 (%)	P value
Retired but not active	27 (36.5)	6 (30.0)	0.150
Retired but active	47 (63.5)	14 (70.0)	
MARITAL STATUS	Male N = 76 (%)	Female N = 24 (%)	P value
Married	70 (92.1)	10 (41.7)	<0.0001
Divorced	0 (0.0)	2 (8.3)	
Widow(er)	6 (7.9)	12 (50.0)	

**Table 2:** Sexual perception in the older adults

	Male	Female	^X2^	P value
Do you still engage in sexual activity?	41 (53.9%)	7 (29.2%)	4.488	0.03
Is sex still pleasurable?	52 (70.3%)	7 (30.4%)	11.685	<0.001
Would you consider it safe to engage in sexual activity?	60 (78.9%)	11 (45.8%)	11.442	0.003
Is engaging in sexual activity a taboo?	4 (5.4%)	8 (33.3%)	13.154	<0.001
Do you have sex related problems?	21 (28.0%)	1 (4.2%)	5.975	0.012
Have you discussed your sexual problems with your partner?	21 (42.0%)	2 (20.0%)	1.706	0.291
Have you discussed your sexual problems with a doctor?	13 (23.2%)	0 (0.0%)	2.891	0.190

## Discussion

This study shows certain misconceptions about sexuality amongst older adults with some gender differences as more men still engaged in sexual activity and considered it safe as well as pleasurable than women. Furthermore, there were overt sexual challenges in the older adults which majority neither disclosed to their spouses nor their attending physicians. Several studies have reported similar gender difference with advancing age that traverses the frequency of sexual activity, sexual satisfaction and desire for sex [3, 11–13]. Despite the misconception about sexuality in this group and persistent sexual needs, they are often overwhelmed by their physical, medical and socioeconomic burdens [14]. In our study, systemic hypertension was the commonest medical condition as a sizeable number of respondents were on regular antihypertensive medications. Similarly, a relatively smaller percentage had medical conditions like diabetes mellitus and arthritis which could affect sexual performance in the respondents. Although some of the respondents used pharmaceutical enhancer for sexual performance, a number of the co-morbid medical conditions and their treatments are known to affect sexuality [15, 16]. Sexually active men reported having sex as safe and culturally acceptable with aging. However, the women were indifferent, less desirous and also considered it prohibited and unsafe. This decline in interest and perception in the older women could be due to the changes in the female anatomy with aging as a sizable number complained of dyspareunia. The hormonal transition with its accompanying low serum level of oestrogen and testosterone is known to be responsible for decreased libido, sensitivity and dyspareunia in older women as genital atrophy and diminished lubrication interfere with sexual comfort and pleasure [4, 17]. Notwithstanding, older women who participated in this study failed to admit to having sexual dysfunction which could be their acceptance of fate and probable consideration of it being the normal irreversible aging phenomenon [18]. Consistent with previous studies, women were less likely than men to discuss sexual challenges with a Physician [6]. This could be due to the failure to initiate this conversation by both parties, discrepancy in age and sex of patient and physician, and the dual negative societal attitudes about sexuality in women and the elderly [19–22]. Similarly, our study showed that women infrequently discussed their sexual challenges with their spouses who could be due to societal perspective to downplay women's sexual desire and to tag the demand for sex in females as promiscuity [22].

Although many older male adults were sexually active, the degree of erectile dysfunction is often considered as an inevitable consequence of aging with its attendant low testosterone level which is likely to be an early indicator of endothelial dysfunction from atherosclerosis and resultant underlying cardiovascular disease. This earlier effect might be seen in the penile arteries due to its smaller diameter than the vessels of the heart and brain [23–25]. Anxiety about their sexual performance was reported in 25% of male respondents which was similar to the 27% in an earlier study [6]. This could be in the bid to impress their spouses in bed and also the feared possibility of a disappointing weak erection as their spouses were relatively younger women. On the contrary, dyspareunia was reported in 25% of the women in our study which was higher than 11.3% reported by Rosen et al [26]. It is however unlikely that pain-pleasure threshold of an African is lower, rather a reflection of willpower, culture of resilience and submission making them endure the pain to satisfy the sexual needs of their husbands [27]. Even though patients’ medical ailments were the most commonly reported reason for sexual inactivity in our study, this was contrary to the findings by Lindau et al where partners’ physical health accounted for the significant sexual inactivity [6]. Women rarely and less frequently initiate sex than their male counterpart, therefore the severity of medical ailment and its likely complications may prevent respondents from engaging in sexual activity as majority of the participants in our study are males [28]. This study is not without some limitations. Being a hospital based study; one might not be able to generalize the findings to the larger community. We, therefore, recommend a community based study into the sexuality of the older adults and the urgent necessity for doctors to take an interest in patients’ sexual history especially the older adults’ whenever they present in the hospital.

## Conclusion

Being old does not exempt one from sexual activity. Therefore, attending physicians must necessarily create a conducive environment to engage the older adults in discussions regarding their sexual health. The older women, particularly in our society, should be enlightened on the need to consciously express concerns about their sexual health.
